# Implementation of national staffing standards in two South African long-term care facilities: a document review

**DOI:** 10.1186/s12913-026-14343-2

**Published:** 2026-05-14

**Authors:** Emerentia C. Nicholson, Mariana M. Van Der Heever, Cornelle Young, Anita S. Van Der Merwe

**Affiliations:** Department of Nursing and Midwifery, Stellenbosch University, Cape Town, South Africa

**Keywords:** Allocation, Caregivers, Long-term care, Nurse staffing, Skill mix

## Abstract

**Background:**

Nurse and caregiver staffing for long-term care facilities in South Africa should be based on national legislative prescriptions regarding the number of all nurse categories and caregivers, the skill mix, and staff allocation aligned to the acuity of residents. This article provides a perspective on understanding the implementation of the national staffing standards in two South African long-term care facilities.

**Methods:**

The larger study comprised a multiple-case study design using multi-methods (document review and qualitative interviews) to explore how such facilities implement nurse and caregiver staffing. Ethical board approval for the study and institutional permission were obtained. This article concerns the findings of the document review conducted in one private for-profit and one state-subsidised long-term care facility, as reviewed from forty-five purposefully selected documents. Sources were triangulated to verify the credibility and representativeness of these documents. ATLAS.ti aided the qualitative analysis of the main themes related to staffing levels, skill mix, and staff allocation. The findings are reported in narrative form.

**Findings:**

The findings showed that fewer nurses were employed, resulting in an overuse of caregivers, as less qualified staff provided the bulk of resident care. An inadequate skill mix may lead to neglecting residents’ acuity levels in staff allocations.

**Conclusions:**

The overreliance on caregivers contribute to an inadequate skill mix and results in the neglect of residents’ acuity levels in care provision. The staffing decisions of CEOs of long-term care facilities should adhere to legislative prescriptions.

**Supplementary Information:**

The online version contains supplementary material available at 10.1186/s12913-026-14343-2.

## Background

The growth in the older population and the expected increase in the number of older persons worldwide intensify the need to proactively plan for the care of older persons in long-term care facilities (LTCFs) [1]. Long-term care encompasses the provision of health care and personal services to older persons by health care professionals over extended periods, supporting them in addressing functional, physical, and cognitive limitations [2, 3]. Different terminology is used for LTCFs internationally, e.g., nursing homes, residential care homes, or skilled nursing facilities. In this article, ‘LTCFs’ refer to all facilities that provide long-term care to older people. International staffing models for LTCFs varied. Neither the World Health Organization (WHO) nor the international Organisation for Economic Co-operation and Development (OECD) mandates specific staffing standards for LTCFs [4, 5]. The WHO recommends using workload indicators to determine staffing based on residents’ needs [4], while the OECD recommends that its member countries determine staffing at the national and facility levels [5]. On the African continent, Ghana has no staffing standards [6], whereas Tanzania and Zimbabwe have minimal guidelines, as institutional care is limited because the traditional culture of Black Africans emphasises care within the family context [2, 7]. In Australia, the Federal Government requires LTCFs to make provision for adequate, qualified, and skilled staff [6, 8], while Germany in Europe, and Canada and the United States in the North American continent have prescribed staffing standards [9–11]. The South African staffing standards, as outlined in the Regulations Regarding Older Persons, 2010, specify the skill mix and staffing levels, stipulating that Category 3 or frail residents receive a minimum of 18 hours of care per week. Category 2, who are not frail but need staff assistance, must receive a minimum of 9 hours of care per week, while Category 1 residents are deemed independent and receive no staff assistance [12]. The hours of care needed per week are expressed as hours per resident day (HPRD). LTCFs use the HPRD to determine the required staffing levels, i.e., the number of all categories of nurses and caregivers needed to provide resident care [13]. HPRD varies between countries. For example, in 2020, United States LTCFs provided an average of 3.53 to 3.89 HPRD [10]. Germany provided more than 4 HPRD [9], compared to South Africa with 2.57 HRPD for Category 3 and 1.29 HPRD for Category 2 residents [12].

In addition to having the required staffing levels, a diverse skill mix is needed, inclusive of different categories of staff with diverse skill sets and expertise [14, 15]. Internationally, the terminology used to describe nurse categories includes registered nurses, advanced practice nurses, licensed practical nurses, and nurse assistants [14, 16]. The South African nursing categories include registered nurses (RNs), enrolled nurses (ENs), and enrolled nursing assistants (ENAs), each with their own scope of practice, which contains the legal parameters within which they may practice. RNs consist of registered professional nurses who provide comprehensive care and registered general nurses who provide general nursing [17]. RNs have either a three-year diploma or a four-year degree-level training. The phased-out EN qualification allowed for two years of training and resultant provision of basic nursing care. However, no new graduates have been trained since the last enrolment for this category on 31 December 2019 [18]. ENAs further provide basic nursing care with a one-year training program (120 credits) that leads to a higher certificate [17, 19]. South African nurses are supported by caregivers, who are not nurses but unlicensed lay workers, assisting residents with their activities of daily living, such as bathing, turning bedridden residents, and feeding [20]. The mandatory skill mix for South African LTCFs is as follows: For Category 3 residents, 33% must be RNs, but 50% of the RNs may be replaced with ENs; thus, at least 16.5% RNs and 16.5% ENs. The remaining 66% of the staff may be ENAs, with caregivers who may replace 50% of this 66%; therefore, there could be 33% ENAs and 33% caregivers. Regarding Category 2 residents, 16% of the staff must be RNs, and they may not be replaced with ENs. Subsequently, very little variation should occur in the mandatory number of RNs for Category 3 and 2 residents (Category 3–16.5%, Category 2–16%). The remaining 84% of staff may be ENAs, of whom caregivers may replace 50% [12].

LTCFs experience staff shortages due to the increase in the number of older persons, a lack of qualified staff, and inflation rates causing price increases of services and goods [21]. Staff shortages lead to inappropriate staff mixes and fewer nurses and caregivers than recommended for LTCFs, especially RNs [9, 10]. South African LTCFs also experience staff shortages with low staffing levels and an inappropriate skill mix [22].

Studies concerning LTCF staffing in South Africa or the African context could not be located. This article reports on the findings of a document review conducted in a South African private for-profit and state-subsidised LTCF to answer the research question on barriers and facilitators to implementing staffing standards. The document review was conducted as part of a larger PhD study, which included a scoping review and a holistic multiple-case study (comprising a document review and interviews) to develop a framework that informs staffing models for LTCFs in resource-constrained contexts. The study was underpinned by Mueller’s ‘Framework for Nurse Staffing in Long-term Care Facilities’, which provides a guide to nurse managers for analysing staffing needs in LTCFs and assists with allocating staff resources according to residents’ needs [23]. The central concepts in Mueller’s framework namely staffing levels, skill mix, and staff allocation based on residents’ acuity guided this study’s data collection and analysis processes. The study was also done from a critical realism perspective. According to Roy Bhaskar, critical realism holds that knowledge exists on three levels or domains: the empirical domain (containing evidence observable to observers), the actual domain (underlying issues that may or may not be observable), and the real domain (causes that are not observable to observers) [24]. The findings of the document review are presented in a narrative format, utilising themes that reflect the central concepts of the study, specifically staffing levels, skill mix, and staff allocation based on residents’ acuity.

## Methods

### Study design

A document review was conducted as it enabled the systematic assessment and interpretation of a range of documents related to how and why LTCFs implemented nurse and caregiver staffing standards.

### Setting

Documents related to nurse and caregiver staffing in LTCFs were collected and reviewed from the public domain (e.g., legislation), as well as from one private for-profit and one state-subsidised LTCF in the Cape Metropole of the Western Cape province, South Africa. The state-subsidised LTCF receives revenue from old-age grants, private pensions, and state subsidies, and provides services in a resource-constrained setting, thus in an environment with limited economic, social, and human resources and infrastructure [25]. The private for-profit LTCF was selected because it represents a median-income rather than a high-income private facility in South Africa. The assumption was that the private for-profit LTCF provided services in a resource-rich setting with adequate resources; however, this facility also experienced nurse shortages, and therefore, it was considered that services were delivered in a resource-constrained setting.

### Population and sampling

Documents were purposefully selected based on whether their context and information on nurse and caregiver staffing brought new insights on how LTCFs implemented staffing standards. The number of documents was not predetermined, but depended on whether the information contained could answer the research question and provide enough depth and richness, to the point that no new themes were identified during the thematic analysis. The sample comprised 45 documents, including eight job descriptions for RNs, ENs, ENAs, and caregivers; 12 monthly duty rosters; minutes from one Annual General Meeting (AGM); minutes of one Board of Directors meeting; six months’ bed lists containing the names of residents; fifteen staff allocation pages; and two quarterly progress reports submitted to the Department of Social Development. Additionally, a Facebook page and a website were reviewed. Public documents selected for review included the Regulations Regarding Older Persons, 2010 [12], the Older Persons Act 13 of 2006 [26], the nurse categories’ scopes of practice [17, 27], and the Western Cape Government’s Health Norms and Standards [20]. Data collection continued until data saturation was reached to the point where the researchers determined that the documents were representative in answering the research question, with no new documents that were encountered to bring insight. Thus, the 45 documents contained sufficient detail about the topic of staffing [28].

### Data collection tool

A data extraction tool (Microsoft Word) was used to capture the collected data. The extraction tool contained detail such as the names of the documents, dates compiled or developed, locations where the documents were found (e.g., online or in the LTCFs’ administrative office), authors of the documents, as well as whether they were primary (e.g., staff allocation book) or secondary sources (e.g., Regulations Regarding Older Persons, 2010).

### Data collection

Appointments were scheduled with the facility CEOs, and copies of the raw data were collected from the LTCFs between December 1st and 29th, 2022. Data from public documents were collected online between April 1 and June 30, 2023. To ensure the anonymity of the LTCFs, the code ‘P1’ was used for the private for-profit and ‘S2’ for the state-subsidised LTCF.

### Trustworthiness

The use of an extraction tool ensured a data trail, enhancing the study’s dependability [29]. Documents containing numerical data, such as duty rosters, were transcribed into a Microsoft Word document. Field notes and reflective comments were captured because the researchers analysed the data through an interpretive lens, thereby recording both the facts the documents conveyed and the possible meanings of the data. Sources were triangulated to verify the credibility and representativeness of documents, for example, by checking whether staff names on the duty rosters matched the list of staff employed and those in the allocation book. The trustworthiness of the LTCFs’ documents was further assessed using documents in the public domain, such as job descriptions, which were then compared with the available nurses’ categories’ scope of practice. The primary author coded the data, with the co-authors who co-coded randomly selected data portions to prevent over- and under-coding and bias, and thus facilitate coder reliability. The reviewed facility-specific documents are not publicly available due to their sensitive nature.

### Data analysis

ATLAS.ti was used to manage the data. In alignment with the study’s underlying paradigm of critical realism [24], the findings of the document review were analysed qualitatively. Braun and Clarke’s (2006) inductive thematic analysis and an inductive coding process were employed. This allowed themes to emerge from the raw data, instead of using a predetermined analytical framework. The researchers analysed the documents (empirical data) iteratively and reflected on their findings. After an initial review of the documents, a deeper analysis of their content helped to identify possible underlying reasons for actions that were not directly visible (real domain). Such a deeper delving into the documents improved the understanding of the content. Codes were created, data sections named, and themes were defined, named, and reviewed to ensure that they captured the study’s key concepts, including staffing levels, skill mix, and aligning of staff allocation with residents’ acuity. Theme saturation was reached when additional coding and theme creation were counterproductive [30].

### Findings

Table 1 presents the five themes and fourteen subthemes developed using a thematic analysis process.

**Table 1:** Themes and subthemes derived from the documents

Themes	Subthemes
Contextual factors shape the implementation of staffing standards	Practices influencing employee wellbeing
	Availability of relief staff
	Shift scheduling was implemented to provide 24-hour care
Nurses and caregivers were left to their own devices	Limited guidelines for nurses and caregivers
	Absence of formal communication structures
Lower nursing and higher caregiver staffing levels	Low nurse-to-resident ratios
	High caregiver-to-resident ratios
	A mismatch between total nurse and caregiver levels
Inequalities in the skill mix	Nurses’ and caregivers’ work experience, competencies, and training
	Proportion of nurses versus caregivers
Neglecting residents’ acuity in staff allocations	Allocation strategies
	Disregarding nurses’ scope of practice and caregivers’ job scopes
	Non-nursing tasks
	Care provision is mostly by caregivers

### Theme 1: Contextual factors shape the implementation of staffing standards

The AGM minutes in P1 reflected that nurses and caregivers received employee benefits, including pension fund contributions. S2’s management did not offer any employee benefits beyond salaries. P1’s duty rosters showed that the staff was divided into two shifts to provide 24 h of care. Caregivers worked 11-hour day shifts, while night shifts lasted 13 h. The RNs’ day shifts ranged from 08:00 to 17:00, and the night shifts from 19:00 to 07:00, with only caregivers on duty without nurse supervision for three hours per day. In S2, nurses and caregivers worked 12-hour shifts, either from 7:00 to 19:00 or from 19:00 to 7:00. P1 did not have nurse vacancies, whereas S2 had vacancies for two ENAs and one EN, but the records showed no attempts to fill these positions over the last six months. Both LTCFs provided relief staff for caregivers when they were absent due to illness or vacation days, but not for nurses. P1 only employed RNs and excluded other subcategory nurses, such as ENs and ENAs. P1’s RN in charge was the only full-time employed RN and was supported by five other RNs working as ‘locums’ to cover the remaining shifts. The locum RNs worked at the facility for between one and five years and could choose to work on demand, thereby covering shifts without requiring P1 to supply relief RNs. In contrast, S2’s inability to provide relief staff for an RN who took two weeks of scheduled vacation leave left the LTCF with only one remaining RN responsible for 101 residents at the facility for two weeks, and no RN coverage over weekends or on any night shifts. No disciplinary measures were recorded in P1; however, disciplinary actions were frequently taken in S2, including issuing warnings to five nurses and two caregivers during the six months preceding data collection. The warnings included issues such as showing a lack of respect towards team members and signing off on medication that was allegedly not administered to residents.

### Theme 2: Nurses and caregivers were left to their own devices

No job-specific policies or procedures were available in the two LTCFs. P1 had no human resource policies, while S2 possessed a generic human resource policy manual. P1’s job descriptions were detailed and included job purposes, required qualifications and experience, and performance expectations. S2’s job descriptions included a list of tasks, such as RNs being required to answer calls, monitor residents’ blood pressure, and allocate staff according to their duty rosters. The ENs in S2 had more comprehensive job descriptions than the RNs and were responsible, among other things, for administering medication and providing staff members with in-service training. The ENAs in S2 were tasked with direct resident care, including wound care, tube feedings, and vital signs monitoring. S2’s caregivers’ job descriptions were similar to those of the ENAs but did not include monitoring residents’ vital signs or assisting with tube feedings. Formal staff meetings did not take place in the two LTCFs. Staff-related issues in P1 (e.g., salary increases) were noted in the minutes from the AGM. In contrast, matters related to the staff in S2, such as a quote for nursing policies and procedures, were reflected in the minutes of one Board of Directors meeting.

### Theme 3: Lower nursing and higher caregiver staffing levels

Table 2 displays the minimum required staffing levels compared to the actual staffing provided in the two LTCFs.

**Table 2:** A comparison of the prescribed staffing levels versus actual staffing levels in the two LTCFs

Categories of residents	Minimum prescribed hours of care per week	(Staffing level includes all categories of nurses and caregivers. Numbers 0.6 and higher are rounded to one.)									
		P1					S2				
		Number of residents in P1	Minimum prescribed staff level for P1	Minimum prescribed HPRD for P1	Actual staff level in P1	Actual HPRD in P1	Number of residents in S2	Minimum prescribed staff level	Minimum prescribed HPRD for S2	Actual staff level in S2	Actual HPRD in S2
Cat. 1	0	0	n/a				27	0			
Cat. 2	9	0	n/a				28	n/a			
Cat. 3	18	35	16				46	n/a			
Combined Cat 2 & 3	13	n/a	n/a				74	24.05			
**Total**		**35**	**16**	**2.57**	**22**	**3.59**	**101**	**24**	**1.86**	**37**	**2.85**

HPRD: Hours per resident day

According to the staffing model outlined in the Regulations Regarding Older Persons, 2010 [12], P1 needed a minimum of 16 staff units, while S2 needed a minimum of 24 staff units (comprising all categories of nurses and caregivers), based on their respective numbers and categories of residents. The calculations use the provided formula: total residents multiplied by hours of care per week, divided by 40 h in a workweek, yielding 15.75 and 24.05 staff units, respectively (rounded to 16 and 24 staff units). However, P1 provided 22 staff units (*N* = 22) and S2 provided 37 (*N* = 37). The minimum prescribed HPRD was derived from the following formula: (total staff units × 40 h workweek / 7 days) / total number of residents. Both LTCFs exceeded the minimum prescribed HPRD. P1 provided an HPRD of 3.59 instead of 2.57, while S2 provided an HPRD of 2.85 instead of 1.86.

The actual nurse staffing level in P1 was limited to one full-time RN and five ‘locum’ RNs who collectively covered four shifts. Thus, P1 had a full-time equivalent of four RNs (*n* = 4) and no ENs or ENAs. The RN-to-resident ratio was 1:35 during both day and night shifts, except for a three-hour period daily when no nurses were on duty. In S2, the nursing staff included two RNs (*n* = 2), seven ENs (*n* = 7), and three ENAs (*n* = 3), with a day shift RN-to-resident ratio of 1:101. Depending on the number of ENs and ENAs available per day shift, the EN ratio varied between 1:33 and 1:50, while the ENA ratio varied between 1:50 and 1:101. Since no RNs were available for the night shift, ENs led the 12-hour night shifts with no other nursing categories on duty, resulting in a ratio of 1:101. Regarding caregiver staffing levels, P1 had 17 full-time caregivers, supplemented by one relief caregiver, resulting in a full-time equivalent of 18 caregivers (*n* = 18). The caregiver-to-resident ratio was 1:6 during the day and 1:12 during the night shifts. In S2, the caregiver staffing levels consisted of 25 caregivers (*n* = 25), with a day shift caregiver-to-resident ratio ranging from 1:11 to 1:13 and a night shift caregiver-to-resident ratio of 1:25. The staffing levels were reached by appointing fewer nurses and increasing the number of caregivers, resulting in an uneven skill mix.

### Theme 4: Inequalities in the skill mix

The RNs in P1 needed to have five to ten years of experience in geriatric care, recent experience in elderly care (without specifying what constitutes ‘recent’), and formal qualifications in dementia, frailty, palliative care, sub-acute care, and rehabilitation care. Caregivers were required to have experience in care settings and training from trustworthy organisations, preferably accredited by the Sector Education and Training Authorities of South Africa. The tasks of RNs and caregivers were linked to key performance indicators. S2 did not specify that caregivers or any category of nurses needed specific work experience, knowledge, skills, or competencies. There were no records of in-service training provided to the staff in the two LTCFs. Table 3 displays the minimum required skill mix versus the actual skill mixes provided in the two LTCFs.

**Table 3:** A comparison of the prescribed skill mix versus the actual skill mix in the two LTCFs

Nurse categories and caregivers	P1						S2					
	Required number of nurses and caregivers	Prescribed skill mix	Prescribed HPRD	Actual nurses and caregivers	Actual skill mix	Actual HPRD	Required number of nurses and caregivers	Prescribed skill mix	Prescribed HPRD	Actual nurses and caregivers	Actual skill mix	Actual HPRD
RNs	3	16.75%	0.43	4	18.1%	0.65	3	12.5%	0.23	2	5.4%	0.15
ENs	3	16.75%	0.43	0	0%	0	3	12.5%	0.23	7	18.9%	0.54
ENAs	5	33.25%	0.86	0	0%	0	9	37.5%	0.70	3	8.1%	0.23
Total nurses	**11**	**66.75%**	**1.71**	**4**	**18.18%**	**0.65**	**15**	**62.5%**	**1.16**	**12**	**32.4%**	**0.82**
Caregivers	5	33.25%	0.86	18	81.81%	2.94	9	37.5%	0.70	25	67.6%	1.93
**Total**	**16**	**100%**	**2.57**	**22**	**100%**	**3.59**	**24**	**100%**	**1.86**	**37**	**100%**	**2.85**

EN: Enrolled nurse; ENA: Enrolled nurse assistant; HPRD: Hours per resident day; RN: Registered nurse

The actual skill mix in South Africa includes RNs, ENs, ENAs, and caregivers. While S2 included all nurse categories and caregivers, P1 only had RNs and caregivers, lacking ENs and ENAs. P1’s skill mix needed to consist of 66.75% nurses and 33.25% caregivers, while S2 had to provide a skill mix of 62.5% nurses and 37.5% caregivers [12]. However, from P1’s total staffing (*N* = 22, 100%) and S2’s total staffing (*N* = 37, 100%), the proportion of nurses in the skill mix was 18.18% and 32.4%, respectively. In contrast, the proportion of caregivers in the total skill mix was 81.81% and 67.6%, respectively. Instead of a minimum total nursing HPRD of 1.71 in P1 and 1.16 in S2, P1 provided 0.65 HPRD and S2 0.82. These findings suggest an overuse of caregivers while neglecting to consider residents’ acuity levels.

### Theme 5: Neglecting residents’ acuity in staff allocations

The RNs’ job descriptions in both facilities indicated that they were responsible for allocating the appropriate staff to residents. However, formal staff-to-resident allocation was not documented in P1. The RNs in S2, or ENs in the absence of RNs, followed an allocation strategy where the nurses and caregivers were allocated to specific geographical areas, such as designated corridors within S2, for approximately three-month periods. The RNs and ENs on night duty were not assigned. Pages from the allocation book included times for tea breaks, lunch, and the corridors to which the staff were assigned. Still, they showed minimal variations with the same information repeated daily. No provisions were made for individual residents or changes in residents’ needs, suggesting that residents’ acuity was not considered when allocating nurses and caregivers to them.

The job descriptions in P1 specified that RNs were required to work within their scope of practice, which included compiling resident care plans, training staff, allocating staff per shift, and adhering to clinical practices by following established policies and procedures. However, records of staff training and allocation were absent, and P1 lacked formal policies and procedures. Caregivers were responsible for non-medical resident care in P1, but they had to assume nursing responsibilities since they worked three hours daily without RNs or other nurse categories on site. In S2, RNs and ENs were assigned for ‘supervision’ and ‘medication’ in ‘all areas’ without explanations or references to residents. Since ENs led the night shifts and some weekend day shifts in the absence of RNs, they had to administer medication without RN supervision beyond their scope of practice [17, 27]. The ENAs were tasked with providing ‘basic care’ without mentioning residents, but they were also required to perform non-nursing tasks, such as laundry. It appeared that caregivers provided the bulk of resident care in both LTCFs. No adverse events were recorded in P1. However, information obtained through participant interviews (findings not reported in this article), provided contradictory information. S2 included several adverse events in the two quarterly reports to the Department of Social Development of South Africa (over six months), including six residents falling, six residents with chafing wounds, one with blisters, one open septic wound, and three residents presenting with discoloured skin.

## Discussion

The 45 documents reviewed as part of a multiple-case study provided an overview of the two LTCFs’ staffing levels and skill mix, and how their task allocations aligned with the nurses’ scope of practice, caregivers’ job descriptions, and residents’ acuity. Mueller (2000) suggested that nurse managers should consider contextual factors when planning for adequate staffing of nurses and caregivers in LTCFs, as contextual factors influence staff management. These contextual factors include detailed job descriptions to guide nurses and caregivers in their duties and responsibilities [23]. The absence of job-specific policies in the LTCFs is concerning, as institutional policies containing updated scientific evidence could guide staff [31], inform their decision-making processes, and should include best practices and delegation levels [32]. Limited formal communication structures existed in the two LTCFs, despite meetings between management, nurses, and caregivers being essential to promote staff well-being, ensure staff felt they belonged and were supported, and facilitate discussions on resident matters, thereby enhancing work performance [33]. Moreover, management undertook frequent disciplinary actions against staff members despite not providing them with job-specific policies and procedures, failing to conduct staff meetings, or documenting the provision of in-service training. Disciplinary procedures are time-consuming and costly, and may adversely affect staff well-being and care delivery [34, 35].

The LTCFs’ failure to provide relief staff for nurses may require the remaining nurses and caregivers to work beyond their scope of practice or job scopes, resulting in care provision that are not aligned with the acuity of the residents. Furthermore, the failure to provide relief staff for nurses may increase the workloads of the remaining nurses and caregivers on duty, impair the remaining nurses’ judgment, leading to substandard care and medication errors [36], and potentially result in even more absenteeism in the LTCFs. Although absenteeism was not analysed in this study, other authors found that absenteeism reflects the well-being of the staff and could be an indicator of LTCFs’ overall performance [37]. Moreover, the nurses’ and caregivers’ shift scheduling indicated that work hours ranged from 9 to 13 h per day. Research has shown that nurses prefer working longer shifts to afford more off days, thereby enabling a better work-life balance, despite the longer work shifts causing fatigue [38]. However, shifts lasting longer than 8 h could result in poorer quality of care and increased safety risks [39, 40].

Whereas the WHO and OECD do not mandate specific staffing models for LTCFs [4, 5], South Africa has explicit staffing standards. Nevertheless, exceeding the minimum required staffing levels for the acuity of residents in this study aligns with the findings of other researchers, indicating that various states within countries or different provinces have chosen to set higher staffing standards than provincial or federal governments have determined [41, 42]. However, higher staffing levels were achieved by employing fewer nurses and more caregivers, which aligns with the global response of LTCFs to managing the perceived difficulties associated with economic restraints and an ageing population. LTCFs appear to replace RNs with nursing assistants to save costs [43, 44], thus delegating care to lower-paid and less-qualified staff [45]. However, the higher HPRD provided by the private for-profit LTCF in this study contradicted typical trends in privately owned LTCFs in the United States, where the private sector appears to have lowered its staffing levels to increase profit margins [46]. In the South African sample, the higher HPRD in the private for-profit LTCF was achieved by over-relying on caregivers, as the skill mix comprised 18.18% nurses and 81.81% caregivers. In comparison, the proportion of caregivers was 81.81%. Private for-profit LTCFs derive income or generate profit by commercialising services to older people, charging fees ranging from R30 000 to R40 000 per resident per month [47]. In comparison, South African state-subsidised LTCFs rely on government subsidies and residents’ social grants for older people of between R2 320 and R2 340 per month [48]. The fee at a state-subsidised LTCF is approximately R8 000 to R12 000 per month. Despite having a lower income than the private for-profit LTCF, the state-subsidised LTCF provided a higher proportion of nurses (32.4%) and caregivers (67.6%) in the skill mix than the private for-profit LTCF.

A study conducted in 43 Italian nursing homes (33 private and 10 public) also revealed trends of utilising less qualified or unlicensed staff to provide direct resident care. At the same time, RNs were more involved in care planning and medication management [49]. Employing fewer nurses and more caregivers results in a low proportion of nurses compared to caregivers, despite differences in the various categories of nurses’ qualifications and skills and those of the caregivers. Authors suggest that, as the care needs of residents increase and become more complex, staff qualifications and experience become increasingly vital when providing resident care [15, 23, 50]. Thus, LTCFs should include more staff with advanced skills and qualifications and emphasise recruiting RNs with degrees rather than diplomas to ensure essential clinical skills in total staffing. Moreover, Mueller et al. mention that RNs are also considered experts in gerontological nursing and play a key role in residents’ care planning [51]. Various studies have found that using fewer high-qualified staff and more unlicensed staff can decrease the quality of resident care [43, 44]. Although the private for-profit LTCF in this study did not document adverse events, the impact of the skill mix on resident outcomes was noticed in the state-subsidised LTCF in this study. The state-subsidised LTCF provided fewer nurses and more caregivers than legislation prescribed, and reported various adverse events, such as residents presenting with septic wounds, blisters, and falls. Higher proportions of nurses in the skill mix, especially RNs, led to less pressure ulcers [52], less falls and use of restraints [44], and lower emergency department visits and rehospitalisation rates [53]. Increasing the number of RNs in the skill mix may enhance the cost-effectiveness of facilities when considering the high costs associated with adverse health outcomes [54]. However, nursing homes appear to struggle with recruiting adequately trained staff [49]. RNs may be reluctant to work in nursing homes due to poor working conditions, a lack of employee benefits [55], poor promotion opportunities, and a high work burden [56].

The study findings, which confirmed that residents’ acuity was not considered when allocating staff, aligned with a study conducted in the Netherlands, where researchers found that residents received the same care regardless of their individual needs [57]. There also seemed to be a disregard for the nurses’ scope of practice when assigning responsibilities, such as ENs in the state-subsidised LTCF administering medicines without RN supervision [17, 27]. Other authors also found that Licensed Practical Nurses (LPNs) adhere better to their scope of practice when their supervisors are informed about the differences in scope of practice, for example, between RNs and LPNs [51]. Caregivers’ roles include assisting residents with their daily activities [20, 53]. However, in this study, caregivers had to assume nursing responsibilities and perform professional tasks in the absence of nurses.

### Strengths and limitations

To our knowledge, this is the first study to explore staffing levels, skill mix, and staff allocation in two South African LTCFs. Although valuable insights were obtained through this document review, it is necessary to acknowledge the study’s limitations. Only two LTCFs in the Western Cape Province were included in the multiple-case study due to cost and time constraints. Transferability of the findings to other geographical contexts may thus be limited. Additionally, the selected LTCF documents were not specifically prepared for a research project and may have lacked relevant information. The absence of staffing frameworks in Africa necessitated the use of Mueller’s framework, which was embedded in a high-income country and may have influenced this study’s findings.

## Conclusion

LTCFs achieved prescribed staffing levels by employing fewer nurses and more caregivers. The overuse of caregivers led to less qualified staff providing the bulk of resident care. As fewer RNs were available, nurses in lower categories were required to work outside their scope of practice. It is recommended that CEOs familiarise themselves with the different scopes of practice for each nurse category to support their nursing staff and prevent them from working beyond their scope. CEOs should also familiarise themselves with the legal implications of using caregivers beyond their job scope. Staff allocation practices did not consider residents’ acuity levels. More research is needed on nurse and caregiver staffing in African LTCFs to strengthen the capacity to care for older persons in a dignified, equitable, and safe environment. Formulation of policies is necessary, including policy initiatives that detail support systems for nurses and caregivers to promote staff well-being and prevent potential burnout. It is imperative to adhere to staffing regulations and ensure that policies and legislation inform the staffing decisions of the LTCFs’ Board of Directors/CEOs. Policy makers and the LTCFs’ Board of Directors/CEOs should also prioritise capacity-building for caregivers, as they make up such a large part of the LTCFs’ workforce. It is also recommended that the caregiver workforce is nationally regulated.

## Supplementary Information

Below is the link to the electronic supplementary material.

Supplementary Material 1

## Data Availability

The datasets supporting the conclusions of this article are included within the article.

## Declarations

### Caregiver

Unlicensed lay workers who are not nurses but work in formal sectors, such as LTCFs. Caregivers assist residents with their activities of daily living, such as bathing, turning bedridden residents, and feeding [20, 26].

### ENAs

Enrolled nursing assistants who provide basic nursing care, such as providing residents with psychosocial and physical support in their activities of daily living under the supervision of an RN [17]. ENAs receive one year of training to obtain a higher certificate (120 credits) [19].

### ENs

Enrolled nurses – ENs provide basic nursing care, such as implementing residents’ nursing care plans and observing residents’ reactions to medication and treatment, under the supervision of an RN [27]. ENs receive two years of training. This is a legacy qualification, and no new graduates have been trained since the last enrollment for this category on 31 December 2019 [19].

### RNs

Registered nurses - RNs consist of registered professional nurses who provide comprehensive care and registered general nurses who provide general nursing [17]. RNs have either a three-year diploma or a four-year degree-level training.

## Abbreviations

CEO: Chief executive officer
ENAs: Enrolled nursing assistants
ENs: Enrolled nurses
HPRD: Hours per resident day
LTCFs: Long-term care facilities
RNs: Registered nurses
