# Supplementary material for: Implementation of national staffing standards in two South African long-term care facilities: a document review
Source: BMC Health Serv Res. 2026 May 14;26:955. doi: 10.1186/s12913-026-14343-2 (PMC13362201; doi:10.1186/s12913-026-14343-2)
Supplement: Supplementary file 1 — Supplementary Material 1 [file 12913_2026_14343_MOESM1_ESM.docx]

**Implementation of staffing standards in two South African long-term facilities: A document review**

**Data extraction tool (extract)**

| **Facility code** | **Data source** | **Document date(s)** | **Location of document(s)** | **Authorship** | **Primary or secondary source** | **Authenticity** | **Credibility** | **Representativeness** |
| --- | --- | --- | --- | --- | --- | --- | --- | --- |
| P1 | Duty rosters | Months of July, August, September, October, November, and December 2022 | Administrative Office | Created by the facility manager. | Primary | The staff's names were typed, and the shifts were completed by hand. The facility manager signed it. | Dates and staff designations on the duty roster correlated with the calendar and employed staff. | The duty roster format for the month included all day and night staff shifts. The roster reflected the shift hours each staff member had to work during each month from July to December 2022, including staff on leave. |
